# Suicide typologies among Medicaid beneficiaries, North Carolina 2014–2017

**DOI:** 10.1186/s12888-022-03741-5

**Published:** 2022-02-10

**Authors:** Josie J. Caves Sivaraman, Shabbar I. Ranapurwala, Scott Proescholdbell, Rebecca B. Naumann, Sandra B. Greene, Stephen W. Marshall

**Affiliations:** 1 Department of Epidemiology, Gillings School of Global Public Health, University of North Carolina at Chapel Hill, Chapel Hill, USA; 2 Injury Prevention Research Center, University of North Carolina at Chapel Hill, Chapel Hill, USA; 3 Present Address: Department of Psychiatry and Behavioral Sciences, Duke University, Durham, North Carolina USA; 4 North Carolina Department of Health and Human Services, Division of Public Health, Injury and Violence Prevention Branch, Raleigh, North Carolina USA; 5 Department of Health Policy and Management, Gillings School of Global Public Health, University of North Carolina at Chapel Hill, Chapel Hill, USA

**Keywords:** Suicide, Firearm, Injury, Epidemiology, Medicaid, Typology, Mental health, Substance use

## Abstract

**Background:**

There is a well-established need for population-based screening strategies to identify people at risk of suicide. Because only about half of suicide decedents are ever diagnosed with a behavioral health condition, it may be necessary for providers to consider life circumstances that may also put individuals at risk. This study described the alignment of medical diagnoses with life circumstances by identifying suicide typologies among decedents. Demographics, stressful life events, suicidal behavior, perceived and diagnosed health problems, and suicide method contributed to the typologies.

**Methods:**

This study linked North Carolina Medicaid and North Carolina Violent Death Reporting System (NC-VDRS) data for analysis in 2020. For suicide decedents from 2014 to 2017 aged 25–54 years, we analyzed 12 indicators of life circumstances from NC-VDRS and 6 indicators from Medicaid claims, using a latent class model. Separate models were developed for men and women.

**Results:**

Most decedents were White (88.3%), with a median age of 41, and over 70% had a health care visit in the 90 days prior to suicide. Two typologies were identified in both males (*n* = 175) and females (*n* = 153). Both typologies had similar profiles of life circumstances, but one had high probabilities of diagnosed behavioral health conditions (45% of men, 71% of women), compared to low probabilities in the other (55% of men, 29% of women). Black beneficiaries and men who died by firearm were over-represented in the less-diagnosed class, though estimates were imprecise (odds ratio for Black men: 3.1, 95% confidence interval: 0.8, 12.4; odds ratio for Black women: 5.0, 95% confidence interval: 0.9, 31.2; odds ratio for male firearm decedents: 1.6, 95% confidence interval: 0.7, 3.4).

**Conclusions:**

Nearly half of suicide decedents have a typology characterized by low probability of diagnosis of behavioral health conditions. Suicide screening could likely be enhanced using improved indicators of lived experience and behavioral health.

**Supplementary Information:**

The online version contains supplementary material available at 10.1186/s12888-022-03741-5.

## Background

Suicide is the 10th leading cause of death in the United States (US), and rates are increasing in every demographic subcategory [1, 2]. Accurate and timely health system identification of people at risk of suicide in order to provide evidence-based clinical interventions is one promising avenue for suicide prevention [3]. Although psychological autopsy studies estimate that 90% of suicide decedents had a diagnosable mental health disorder at the time of death [4], behavioral health disorders and other conditions (such as chronic pain) in patients who progress to suicide are likely underdiagnosed. Even though suicide decedents are at least twice as likely as the general population to have a health care encounter in the 90 days prior to death [5], only about half are ever diagnosed with a mental health condition [2, 6]. Looking beyond diagnostic data may give practitioners a more complete view of patients’ suicide risk.

The Stress-Diathesis model of suicide explains that suicidal behavior is often the outcome of a confluence of distal risk factors confronting one or more proximal stressors which combine to overwhelm the individual’s ability to cope [7]. Providers are most likely to identify suicide risk in the subset of patients with long term, persistent, or recurring mental health problems. However, patients’ experiences of acute mental health crises and stressors related to life events (i.e. the death of a friend or family member, an intimate partner problem, legal problems, etc.) also contribute to suicide risk but maybe less identifiable. Recognizing this, the Zero Suicide model for suicide prevention recommends that clinicians screen for and address suicide risk at every patient interaction [3]. However, patients who are not already accustomed to discussing mental health struggles with their providers may be resistant to such conversations. Asking such patients about life events, in addition to their mood, may be informative.

Some health systems have begun to explore the frontier of linking health record information with other data sources (e.g. mortality data) to improve suicide risk prediction [8–10]. In one recent example [11], authors not only linked to mortality data, but also included small area geocode indicators of neighborhood deprivation and fragmentation, acknowledging that social/circumstantial factors likely also play a role in suicide risk. The current study explores how data on individual-level circumstances may improve clinical suicide screening by describing how suicide decedents’ behavioral health diagnoses align with stressful life events and other experiences.

This study retrospectively linked claims data from a Medicaid population to violent death surveillance data for the purpose of identifying typology classifications (hereafter, “classes”) that characterize patients’ life and health care experiences. We limited all analyses to adults aged 25–54 to create a group with similar health care experiences, so that our results would not be influenced by age-related heterogeneity. We analyzed data separately for male and female suicide decedents because of well-known differences by sex in the prevalence of suicide [1]. Behavioral health disorders are also an important component of our model which are known to be more commonly diagnosed among women [12]. In addition, we explored how class membership was associated with race because suicide is perceived to be less common among Black people, and may also reflect distinct risk factor distributions [13]. Finally, we explored how class membership was associated with suicide method, because people (particularly men) who die from firearm suicide are less likely to have behavioral health diagnoses than those who die from other suicide methods [14].

## Methods

This study characterized suicide decedents using a) data collected from administrative death investigations by law enforcement and medical examiners and b) Medicaid claims. In 2020, the study linked North Carolina (NC) Medicaid claims to suicide decedents who were identified in the NC Violent Death Reporting System (NC-VDRS) as having died between January 1, 2014 and December 31, 2017. The linkage used exact matches on name, date of birth, and sex and was performed by an honest broker so that the authors received fully anonymized data. The NC-VDRS is a component of the National Violent Death Reporting System (NVDRS), which is a national surveillance system for intentional fatalities created by the Centers for Disease Control and Prevention.

### Population

The study population consisted of NC Medicaid beneficiaries aged 25–54 years who died as a result of suicide between January 1, 2014 and December 31, 2017 and were covered for at least 12 continuous months prior to death. This age range was selected due to age-based heterogeneity particularly in lived experiences and suicide means among the youngest and oldest decedents. “Covered” individuals met the age requirement and were enrolled in Medicaid under family planning for low-income people, managed care for behavioral health services, Medicare-Aid, NC Health Choice (coverage for children), or general Medicaid. Demographic variables (age, race, and sex) were defined using NC Medicaid member files. Race was categorized as Black/non-Black because of the perception that suicide is uncommon in this population [13], and because of structural and cultural barriers to behavioral health services impacting this population [15]. Suicide decedents who did not have any circumstance information endorsed were excluded (*n* = 4).

### Measures

We identified typologies to describe the decedents based on characteristics in four domains: Suicidal Behavior, Stressful Life Events, Perceived Behavioral Health, and Diagnosed Behavioral Health (Table 1). The first three of these domains were ascertained from NC-VDRS data abstracted from official post-mortem death investigations by law enforcement and medical examiners. These were composed of 12 specific variables, which were selected from a larger group of NVDRS circumstance variables abstracted from narratives written by law enforcement and medical examiners. Theoretical and/or evidence-based association with suicide are the basis for all circumstance data collected by NVDRS. Each specific circumstance variable is coded as present/not present by highly trained data abstractors. All abstractors undergo a rigorous training process and their work is subject to a re-abstraction of a random sample. Full details of the data quality processes in NVDRS are available [16]. The 12 specific circumstance variables selected for this study were chosen because of their frequencies suggested they would be informative (other circumstances had very low frequencies). The information sources for these reports includes family members and acquaintances of the decedent, witnesses, and/or suicide notes. 99% of cases had at least one known circumstance; cases where this data was missing were excluded from the analysis (*n* = 4). Suicide method (firearm/not firearm) was also based on the information abstracted by NC-VDRS data collectors.

**Table 1:** Domains and indicator variables for suicide latent class analysis

Source	Domain	Concept Measured	Operational Definition
Medical Examiner and Law Enforcement Reports (NC-VDRS)	Stressful Life Events	Death of family member or friend	Considered to have contributed to the suicide
		Intimate partner problem	Considered to have contributed to the suicide
		Family problem	Considered to have contributed to the suicide
		Legal problem (civil/criminal)	Considered to have contributed to the suicide
		Physical health problem	Considered to have contributed to the suicide
	Suicidal Behavior	History of suicide attempt	Lifetime
		Disclosed intent to suicide	Within the last month
		Known to have experienced suicidal thoughts	Lifetime
	Perceived Behavioral Health	Substance use problem	An acquaintance knew this to be a perceived problem at time of death
		Alcohol use problem	An acquaintance knew this to be a perceived problem at time of death
		Depressed mood	An acquaintance knew this to be a perceived problem at time of death
		Mental health problem	An acquaintance knew this to be a diagnosed problem (at any point) which had not resolved by the time of death
Diagnoses and Encounters (NC Medicaid claims)	Diagnosed Behavioral Health	Drug use disorder^a^	Any diagnosis within past 2 years
		Alcohol use disorder^a^	Any diagnosis within past 2 years
		Bipolar disorder^a^	Any diagnosis within past 2 years
		Anxiety disorder^a^	Any diagnosis within past 2 years
		Depressive disorder^a^	Any diagnosis within past year
		Encounter in past 90 days	Any claim reflecting an inpatient or outpatient encounter within past 90 days

*NC-VDRS* North Carolina Violent Death Reporting System

^a^Diagnoses were defined using algorithms from the Centers for Medicaid and Medicare Services

The 4th domain (“Diagnosed Behavioral Health”) was derived from NC Medicaid claims and included behavioral health diagnoses that were referenced in the 12–24-month period prior to death (Table 1). We defined the behavioral health diagnoses using algorithms developed by the Centers for Medicaid and Medicare Services [17]. This 4th domain also included an indicator variable for whether or not the decedent had any encounter with the health care system (outpatient or inpatient) in the 90 days prior to death.

### Statistical analysis

Multivariate analyses utilized a latent class analysis of the 12 indicator variables (Domains 1–3) from NC-VDRS and 6 (Domain 4) from NC Medicaid (Table 1). Latent class analysis is a form of mixture modeling that is used to illuminate unobserved (“latent”) groups within populations. These groups are often referred to as typologies or classes. In this case, we sought to illuminate latent typologies of suicide decedents based on the overlay of their medical diagnoses and life events. To identify the optimal number of classes to extract, we generated up to seven class solutions for each sex separately and evaluated each one based on several model performance criteria (Tables S1 and S2), with emphasis on the Bayesian Information Criterion, which balances class differentiation and parsimony [18]. We described the resulting two-class solutions for men and women based on domain characteristics and univariate associations of race (Black/non-Black) and suicide method (firearm/non-firearm) with class membership, using the 1-Step Approach [19]. In this approach, covariates are added directly to the latent class model as predictors. A limitation of this approach is that if a predictor has a direct relationship to the indicator variables that is not described through suicide class then the inclusion of the predictor can substantively distort typology characteristics. To address this potential issue, we compared indicator variable distributions for the models with and without each predictor, ensuring that the predictors did not create significant classification changes. This work was reviewed by the University of North Carolina at Chapel Hill Institutional Review Board. Analyses were performed in R version 4.0.3.

## Results

Between January 1, 2014 and December 31, 2017, there were 328 suicides among 25–54 year old Medicaid enrollees who were enrolled for at least 12 months prior to death and who met inclusion criteria. Of these, 47% (*n* = 153) were women (women comprise the majority of Medicaid enrollees in this age bracket). The majority of male and female suicide decedents were White (88.3%) with a median age of 41. In the Stressful Life Events domain, men were characterized by a higher likelihood of legal (male: 16.0%; female: 7.8%) and intimate partner problems (male: 25.1%; female: 16.3%; Table 2). In the Suicidal Behavior domain, women were more likely to have had one or more past suicide attempts (male: 22.9%; female: 31.4%). In the Perceived Behavioral Health domain, men were more likely to be perceived as depressed (male: 28.0%; female: 20.9%). Other variables in this domain were either similar between sexes or more prevalent in women. In the Diagnosed Behavioral Health domain, diagnoses were more prevalent in women, with the exception of alcohol use disorder (male: 18.3%; female: 11.8%), and drug use disorder (male: 23.4%; female: 20.3%). Over 70% of decedents had at least one inpatient or outpatient encounter in the 90 days prior to death (73% for men; 80% for women; Table 2).

**Table 2:** Characteristics of female and male suicide decedents age 25–54 (North Carolina Medicaid, 2014–2017)

	Males (***N*** = 175) n (%)	Females (***N*** = 153) n (%)
**Age (med, IQR)**	42 (33, 48)	40 (33, 46)
**Race**		
Black	19 (10.9)	11 (7.2)
White	146 (83.4)	140 (91.5)
Other	<10^b^	<10^b^
**Supplemental Security Income**		
Yes	65 (37.1)	41 (26.0)
No	110 (62.9)	112 (73.2)
**Stressful Life Events**^**a**^		
Death of friend or family	11 (6.3)	<10^b^
Criminal or civil legal problem	28 (16.0)	12 (7.8)
Intimate partner problem	44 (25.1)	25 (16.3)
Family relationship problem	24 (13.7)	17 (11.1)
Physical health problem in the past month	46 (26.3)	33 (21.6)
**Suicidal Behavior**^**a**^		
Recently disclosed suicide intent	54 (30.9)	42 (27.5)
History of suicide attempt (1 or more)	40 (22.9)	48 (31.4)
History of suicidal thoughts	77 (44.0)	65 (42.5)
**Perceived Behavioral Health**^**a**^		
Non-alcohol substance use problem	54 (30.9)	63 (41.2)
Alcohol problem	29 (16.6)	20 (13.1)
Depressed mood	49 (28.0)	32 (20.9)
Mental health problem	118 (67.4)	125 (81.7)
**Diagnosed Behavioral Health**		
Drug use disorder diagnosis	41 (23.4)	31 (20.3)
Alcohol use disorder diagnosis	32 (18.3)	18 (11.8)
Bipolar disorder diagnosis	33 (18.9)	54 (35.3)
Anxiety diagnosis	81 (46.3)	88 (57.5)
Depression diagnosis	58 (38.9)	91 (59.5)
Chronic pain, fibromyalgia, sleep disorders diagnosis	61 (34.9)	59 (38.6)
Any inpatient or outpatient encounter in the last 90 days	128 (73.1)	122 (79.7)
**Suicide Method**		
Firearm	74 (42.3)	42 (27.5)
Poisoning	35 (20.0)	79 (51.6)
Hanging/strangulation	44 (25.1)	27 (17.7)
Other (i.e. fall, drowning, sharp instrument)	22 (12.6)	<10^b^

^a^Variables from the North Carolina Violent Death Reporting System; ^b^Counts < 10 are suppressed

**Fig. 1:**
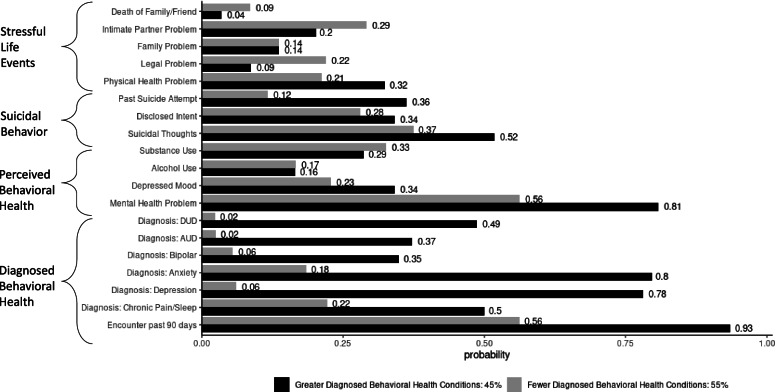
Male class profiles for suicide decedents age 25–54 (North Carolina Medicaid, 2014–2017). DUD = drug use disorder; AUD = alcohol use disorder; the “Greater Diagnosed Behavioral Health Conditions” bars describe the patient typology that was more likely to have behavioral health diagnosis(es) than the “Fewer Diagnosed Behavioral Health Conditions” typology. The two typologies are similar in terms of Stressful Life Events, Suicidal Behavior, and Perceived Behavioral Health

**Fig. 2:**
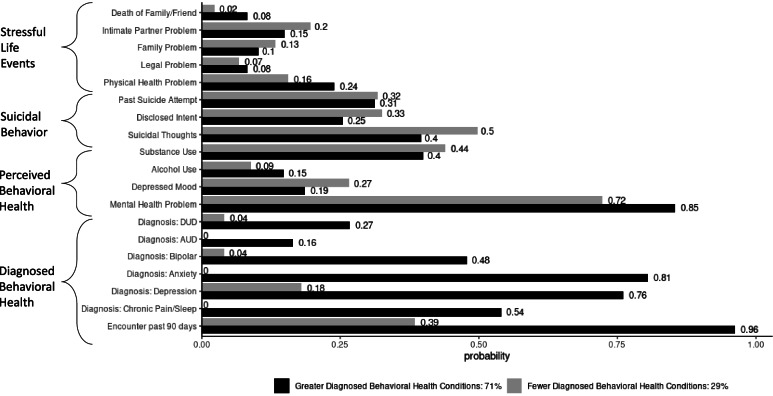
Female class profiles for suicide decedents age 25–54 (North Carolina Medicaid, 2014–2017). DUD = drug use disorder; AUD = alcohol use disorder; the “Greater Diagnosed Behavioral Health Conditions” bars describe the patient typology that was more likely to have behavioral health diagnosis(es) than the “Fewer Diagnosed Behavioral Health Conditions” typology. The two typologies are similar in terms of Stressful Life Events, Suicidal Behavior, and Perceived Behavioral Health

Latent class models suggested a two-class solution for both males and females (eTables 1 and 2). The two classes similarly reflected low to medium probabilities for the indicator variables in the first three domains of Stressful Life Events, Suicidal Behavior, and Perceived Behavioral Health for both men and women (Figs. 1 and 2). For both men and women, the classes diverged in the fourth domain of Diagnosed Behavioral Health, with one class representing higher probability of behavioral health diagnoses (0.35–0.80 in men; 0.16–0.81 in women) and the other lower probability of behavioral health diagnoses (0.02–0.22 in men; 0.0–0.18 in women). We interpreted the two classes as approximating two clusters of patient groups with fewer (hereafter: less-diagnosed) and greater (hereafter: more-diagnosed) Diagnosed Behavioral Health Conditions (Figs. 1 and 2).

Over half of male decedents (55%) and nearly one-third (29%) of female decedents belonged to the less-diagnosed class (Figs. 1 and 2). In the Stressful Life Events domain, both classes of men had slightly higher probabilities than the female classes of experiencing the individual stressors. Men also exhibited somewhat more divergence between the classes in this domain, particularly in legal problems (0.22 in less-diagnosed vs 0.09 in more-diagnosed). In the Suicidal Behavior domain, the less-diagnosed class of men reflected a lower probability of history of attempted suicide (0.12) relative to the more-diagnosed class of men (0.32). These probabilities were similar in the female classes (0.32 and 0.31, respectively). Perceived Behavioral Health was also similar across the male and female classes. Men and women in the more-diagnosed class had very high probabilities (0.81 and 0.85, respectively) of having a mental health problem, according to law enforcement and/or medical examiner reports. These probabilities for the less-diagnosed classes were lower for men in particular (0.56), as well as women (0.72). The classes diverged substantially in the Diagnosed Behavioral Health domain, with the more-diagnosed class exhibiting a higher probability of an inpatient or outpatient visit in the 90 days prior to death for both men (less-diagnosed: 0.56; more-diagnosed: 0.93) and women (less-diagnosed: 0.39; more-diagnosed: 0.96).

Finally, we used odds ratios (ORs) to quantify the likelihood of class membership based on race and suicide method (note that ORs refer to the odds of class membership, not the odds of suicide). Black men and women were more likely to belong to the less-diagnosed class (OR for men: 3.1; 95% confidence interval [CI]: 0.8, 12.4; OR for women: 5.0, 95% CI: 0.9, 31.2) compared to men and women in the White or other race category. However, these estimates were imprecise due to low counts of Black individuals, particularly among women. Men who died from self-inflicted firearm injury were also moderately more likely to be represented in the less-diagnosed class (OR: 1.6, 95% CI: 0.7, 3.4), as compared to men who died from a self-inflicted non-firearm injury. There was no evidence of such an association in women (OR: 0.9, 95%CI: 0.3, 2.9).

## Discussion

This study used latent class analysis to characterize suicide decedents in a Medicaid population using both diagnostic data from claims, and life circumstance data obtained from law enforcement and medical examiner’s reports. For both male and female decedents, two classes were identified. The classes appeared generally similar across domains of Stressful Life Events, Suicidal Behavior, and Perceived Behavioral Health but diverged in their probabilities of being diagnosed with behavioral health conditions.

### Sex

Males had a high probability of being in the class less-diagnosed with behavioral health conditions (55%). In females, the probability of being classified as less-diagnosed was considerably lower (29%). This is consistent with what is known about the higher burden of behavioral health conditions and increased willingness to use mental health services among women [12]. We also found that men who used a firearm for suicide tended to be over-represented in the less-diagnosed class. This is consistent with past findings [14] and may reflect that firearm suicide deaths may be characterized by a greater degree of impulsivity [20]. Because firearms are the most lethal means of inflicting self-injury [21], a person who impulsively attempts suicide with a firearm is much more likely to die as a result of the attempt than if they had made the attempt using any other method. In the latter scenario, the person may have been more likely to receive a diagnosis and treatment, preventing a potential re-attempt. Approaches such as counseling on access to lethal means (CALM) may be appropriate to identify and protect people at risk of suicide even in the absence of behavioral health diagnoses [22]. This approach helps patients and family members recognize and form plans about the storage of items that can be used to inflict lethal force.

It is encouraging that the majority of Medicaid suicide decedents (men: 73%; women: 80%) had at least one outpatient or inpatient healthcare encounter in the 90 days prior to death. This indicates that there is opportunity to intervene on these individuals if they can be identified based on their healthcare and life event profiles. Linkage of multiple rich sources of data to allow for better prediction of suicide risk and help connect high risk patients with suicide prevention programs is an area of active research [23].

### Race

The finding of possible over-representation of Black people in the less-diagnosed class for both men and women is concerning. Prior research has documented under-diagnosis of depression and other mental health disorders in Black and other minority populations [15, 24, 25]. Some depression screening tools may exhibit racial bias [15, 26], or providers may not be culturally competent to recognize depressive symptoms in patients who do not share their race [24]. Socioeconomic status, access to care, help-seeking behaviors, and mistrust of the health care system are other possible explanations for this disparity [15]. When disorders are identified, Black people are also less likely to receive consistent treatment [27]. Our finding that Black people were more likely to be represented in the less-diagnosed group may reflect this implicit bias in the health care system and underscores the need for increased racially competent suicide screening and mental health instrumentation and services [28].

There are several possible explanations for the findings that the two classes of patients identified in this study were similar in their life circumstance profiles, yet very dissimilar in their diagnostic profiles. One possible explanation is that the less-diagnosed class did have underlying behavioral health conditions but tended to have less contact with health care providers, meaning behavioral conditions were not identified as current diagnoses in claims data. The Medicaid population is vulnerable to factors that impact continuity of care, including housing and employment instability and reduced family support [29]. Further, for men and women in the less-diagnosed class, it is possible that the apparent incongruity between the NC-VDRS “mental health problem” variable (according to law enforcement/ medical examiner data) and the Medicaid diagnosis variables for mental health disorders represents people who were diagnosed at some point but were not in active treatment. Regardless, even in the less-diagnosed class, the probability for contact with the health system 90 days prior to death was 0.56 among men, and 0.39 among women, suggesting that the low probability of a behavioral health diagnosis in this class is not fully explained by a lack of contact with health care providers.

A second possible explanation is that patients who had an underlying behavioral health condition were in contact with the health system but did not receive a diagnosis. The type of specialty care sought by patients likely varies by sex and is also impacted by having a history of non-suicidal self-injury and suicide attempt, which also vary by sex [30]. These undoubtedly affect whether or not a patient is screened for suicide risk and/or diagnosed with a behavioral health condition (e.g., a visit to a psychiatric clinic is more likely to result in screening/diagnosis than a cardiology visit). Thus, type of health care utilization may partially explain why the proportion classified into the less-diagnosed class was higher in men than women.

A third possible explanation for this study’s finding of similarities in life circumstance profiles, but dissimilar diagnostic profiles, is that the lack of mental health disorder diagnoses in the less-diagnosed class could reflect a true absence of underlying conditions. Mental health disorders have long been considered one of a number of risk factors for suicidal behaviors, but other risk factors have been established, including genetic predisposition, early life experiences, cognitive and personality characteristics, stressful life events, access to lethal means, and others [31–36]. These also likely interact within the context of additional societal and environmental factors [37]. Increasingly robust screening for a wide range of risk factors (including lived experiences) may help identify additional patients. More research into this approach is warranted [38].

This study had several strengths. Linkage between Medicaid and violent death data is unique in the literature and allowed us to examine circumstantial risk factors of suicide that are not captured in claims. The Medicaid population has a high prevalence of many of these risk factors and represents a population that is engaged in healthcare and knowledgeable of public programs, and thus might be amenable to suicide prevention interventions. This study also has several limitations. Because the life circumstance data from NC-VDRS was collected post-mortem, it is prone to recall effects that could lead to over-ascertainment of some items, and there may be variation between death investigators in their ascertainment of life circumstance information. However, NC was one of the first states to implement NVDRS, and NC program administrators have continually striven to improve the quality and consistency of NC-VDRS since the NC program was initiated in 2004. Mental health conditions that were not associated with a Medicaid claim would not be detected by this data source. It is also possible that patients in this study were identified as high risk for suicide but did not receive a behavioral health diagnosis due to stigma or other factors (e.g., implicit racial bias), even if they did receive prevention services. Finally, any population-based suicide study has the potential for under ascertainment of suicide by local death investigation authorities. To address this, NVDRS draws death determinations from three sources (death certificate, medical examiner, and law enforcement).

## Conclusions

Behavioral health diagnosis and treatment are foundational to our health system’s approach to suicide prevention. This analysis indicates that men and women suicide decedents who were Medicaid beneficiaries can be divided into two distinct classes: one with higher probability and one with lower probability of receiving a behavioral health diagnosis prior to suicide. These two classes appear otherwise similar in terms of life circumstances. The class with lower probability of receiving a behavioral health diagnosis may comprise patients with underlying conditions who were not adequately identified diagnostically as high risk for suicide, yet did in fact progress to suicide.

It appeared that having Black race (men and women) and use of a firearm for suicide (men only) were associated with membership in the class with lower diagnostic probability. These findings may be related to implicit racial bias in provision of health care services and a higher degree of impulsivity associated with use of firearms as a means of suicide. Encouragingly, over 70% of decedents had a health system encounter in the 90 days prior to death. Additional screening (including culturally or racially appropriate methodologies) and improved health care-based interventions may help to identify and treat underlying conditions and mitigate chronic and proximal risk factors for suicide.

## Supplementary Information

**Additional file 1: eTable 1.** Class solution criteria for female Medicaid beneficiaries who died of suicide (*n* = 153). A two-class solution was selected. **eTable 2.** Class solution criteria for male Medicaid beneficiaries who died of suicide (*n* = 175). A two-class solution was selected.

## Data Availability

The data that support the findings of this study are available from The North Carolina Department of Health and Human Services and from the Centers for Disease Control and Prevention, but restrictions apply to the availability of these data, which were used under license for the current study, and so are not publicly available. Data are however available from the authors upon reasonable request and with permission of The North Carolina Department of Health and Human Services and the Centers for Disease Control and Prevention.

## Abbreviations

CALM: Counseling on access to lethal means
NC: North Carolina
NC-VDRS: North Carolina Violent Death Reporting System
NVDRS: National Violent Death Reporting System
OR: Odds ratio
US: United States

## References

[1] Ivey-Stephenson AZ, Crosby AE, Jack SPD, Haileyesus T, Kresnow-Sedacca MJ (2017). Suicide trends among and within urbanization levels by sex, race/ethnicity, age group, and mechanism of death - United States, 2001-2015. MMWR Surveill Summ.

[2] Stone DM, Simon TR, Fowler KA, Kegler SR, Yuan K, Holland KM (2018). Vital signs: trends in state suicide rates - United States, 1999-2016 and circumstances contributing to suicide - 27 states, 2015. MMWR Morb Mortal Wkly Rep.

[3] Stanley B, Mann JJ (2020). The need for innovation in health care systems to improve suicide prevention. JAMA Psychiatry.

[4] Cavanagh JT, Carson AJ, Sharpe M, Lawrie SM (2003). Psychological autopsy studies of suicide: a systematic review. Psychol Med.

[5] Ahmedani BK, Westphal J, Autio K, Elsiss F, Peterson EL, Beck A (2019). Variation in patterns of health care before suicide: a population case-control study. Prev Med.

[6] Ahmedani BK, Simon GE, Stewart C, Beck A, Waitzfelder BE, Rossom R (2014). Health care contacts in the year before suicide death. J Gen Intern Med.

[7] Hawton K, van Heeringen K (2009). Suicide. Lancet.

[8] Kessler RC, Hwang I, Hoffmire CA, McCarthy JF, Petukhova MV, Rosellini AJ (2017). Developing a practical suicide risk prediction model for targeting high-risk patients in the Veterans health Administration. Int J Methods Psychiatr Res.

[9] Simon GE, Johnson E, Lawrence JM, Rossom RC, Ahmedani B, Lynch FL (2018). Predicting suicide attempts and suicide deaths following outpatient visits using electronic health records. Am J Psychiatry.

[10] Sivaraman JJC, Naumann RB. Estimating the association between mental health disorders and suicide: a review of common sources of bias and challenges and opportunities for US-based research. Curr Epidemiol Rep. 2020;7(4):352-62. 10.1007/s40471-020-00250-5. 10.1007/s40471-020-00250-5 PMC8092021 33948425

[11] Kessler RC, Bauer MS, Bishop TM, Demler OV, Dobscha SK, Gildea SM (2020). Using administrative data to predict suicide after psychiatric hospitalization in the veterans health administration system. Front Psychiatry.

[12] Kuehner C (2017). Why is depression more common among women than among men?. Lancet Psychiatry.

[13] Joe S, Niedermeier DM (2008). Social work research on African Americans and suicidal behavior: a systematic 25-year review. Health Soc Work.

[14] Sivaraman JJ, Greene SB, Naumann RB, Proescholdbell S, Ranapurwala SI, Marshall SW (2022). Association between medical diagnoses and suicide in a Medicaid beneficiary population, North Carolina 2014-2017. Epidemiology.

[15] Shao Z, Richie WD, Bailey RK (2016). Racial and ethnic disparity in major depressive disorder. J Racial Ethn Health Disparities.

[16] Blair JM, Fowler KA, Jack SP, Crosby AE (2016). The national violent death reporting system: overview and future directions. Inj Prev.

[17] Chronic conditions data warehouse: The Centers for Medicare and Medicaid Services; Available from: https://www2.ccwdata.org/web/guest/condition-categories. Accessed 1 Dec 2020.

[18] Tein J-Y, Coxe S, Cham H (2013). Statistical power to detect the correct number of classes in latent profile analysis. Struct Equ Modeling.

[19] Bolck A, Croon M, Hagenaars J (2004). Estimating latent structure models with categorical variables: one-step versus three-step estimators. Polit Anal.

[20] Sivaraman M, Fahmie TA (2020). A systematic review of cultural adaptations in the global application of ABA-based telehealth services. J Appl Behav Anal.

[21] Elnour AA, Harrison J (2008). Lethality of suicide methods. Inj Prev.

[22] Sale E, Hendricks M, Weil V, Miller C, Perkins S, McCudden S (2018). Counseling on Access to Lethal Means (CALM): an evaluation of a suicide prevention means restriction training program for mental health providers. Community Ment Health J.

[23] Pence BW, Ranapurwala SI. Innovations in suicide prevention research (INSPIRE): University of North Carolina at Chapel Hill. Rockville: National Institute of Mental Health; 2020.

[24] Snowden LR (2003). Bias in mental health assessment and intervention: theory and evidence. Am J Public Health.

[25] Lewis TT, Cogburn CD, Williams DR (2015). Self-reported experiences of discrimination and health: scientific advances, ongoing controversies, and emerging issues. Annu Rev Clin Psychol.

[26] Akinhanmi MO, Biernacka JM, Strakowski SM, McElroy SL, Balls Berry JE, Merikangas KR (2018). Racial disparities in bipolar disorder treatment and research: a call to action. Bipolar Disord.

[27] Bailey RK, Blackmon HL, Stevens FL (2009). Major depressive disorder in the African American population: meeting the challenges of stigma, misdiagnosis, and treatment disparities. J Natl Med Assoc.

[28] Frazer E, Mitchell RA, Nesbitt LS, Williams M, Mitchell EP, Williams RA (2018). The violence epidemic in the African American community: a call by the National Medical Association for comprehensive reform. J Natl Med Assoc.

[29] Mechanic D (2014). More people than ever before are receiving behavioral health care in the United States, but gaps and challenges remain. Health Aff.

[30] Hamza CA, Stewart SL, Willoughby T (2012). Examining the link between nonsuicidal self-injury and suicidal behavior: a review of the literature and an integrated model. Clin Psychol Rev.

[31] Dwivedi Y. The neurobiological basis of suicide. Boca Raton: CRC press; 2012. 23035294

[32] Dube SR, Anda RF, Felitti VJ, Chapman DP, Williamson DF, Giles WH (2001). Childhood abuse, household dysfunction, and the risk of attempted suicide throughout the life span: findings from the adverse childhood experiences study. JAMA.

[33] Felitti VJ, Anda RF, Nordenberg D, Williamson DF, Spitz AM, Edwards V (1998). Relationship of childhood abuse and household dysfunction to many of the leading causes of death in adults: the adverse childhood experiences (ACE) study. Am J Prev Med.

[34] Giner L, Blasco-Fontecilla H, De La Vega D, Courtet P (2016). Cognitive, emotional, temperament, and personality trait correlates of suicidal behavior. Curr Psychiatry Rep.

[35] Qin P, Agerbo E, Mortensen PB (2003). Suicide risk in relation to socioeconomic, demographic, psychiatric, and familial factors: a national register-based study of all suicides in Denmark, 1981-1997. Am J Psychiatry.

[36] Wiebe DJ (2003). Homicide and suicide risks associated with firearms in the home: a national case-control study. Ann Emerg Med.

[37] Turecki G, Brent DA, Gunnell D, O’Connor RC, Oquendo MA, Pirkis J (2019). Suicide and suicide risk. Nat Rev Dis Primers.

[38] Hjelmeland H, Knizek BL (2010). Why we need qualitative research in suicidology. Suicide Life-Threat Behav.

