# Supplementary material for: Suicide typologies among Medicaid beneficiaries, North Carolina 2014–2017
Source: BMC Psychiatry. 2022 Feb 10;22:104. doi: 10.1186/s12888-022-03741-5 (PMC8832648; doi:10.1186/s12888-022-03741-5)
Supplement: Supplementary file 1 — Additional file 1: eTable 1. Class solution criteria for female Medicaid beneficiaries who died of suicide (n = 153). A two-class solution was selected. eTable 2. Class solution criteria for male Medicaid beneficiaries who died of suicide (n = 175). A two-class solution was selected. [file 12888_2022_3741_MOESM1_ESM.docx]

**eTable 1. Class solution criteria for female Medicaid beneficiaries who died of suicide (n=153). A two-class solution was selected.**

| **Class solution** | **Parameters** | **Log likelihood** | **Entropy*** | **AIC*** | **BIC*** | **CAIC*** | **ssBIC*** | **CLC*** | **ICL BIC*** | **NEC*** |
| --- | --- | --- | --- | --- | --- | --- | --- | --- | --- | --- |
| **1** | 19 | -1502.54 | 0.00 | 3043.08 | 3100.66 | 3119.66 | 3040.52 | 3005.08 | 3100.66 | 1.00 |
| **2** | 39 | -1416.57 | 12.11 | 2911.15 | **3029.34** | **3068.34** | 2905.90 | 2857.37 | **3053.56** | 0.14 |
| **3** | 59 | -1366.07 | **20.57** | 2850.13 | **3028.93** | 3087.93 | 2842.19 | 2773.27 | 3070.07 | 0.15 |
| **4** | 79 | -1335.34 | 14.00 | 2828.68 | 3068.09 | 3147.09 | 2818.05 | 2698.68 | 3096.09 | 0.08 |
| **5** | 99 | -1303.81 | 17.08 | 2805.62 | 3105.63 | 3204.63 | 2792.29 | 2641.78 | 3139.79 | 0.09 |
| **6** | 119 | -1281.48 | 18.44 | **2800.95** | 3161.57 | 3280.57 | **2784.93** | 2599.83 | 3198.45 | 0.08 |
| **7** | 139 | -1264.86 | 17.10 | 2807.71 | 3228.94 | 3367.94 | 2789.00 | **2563.91** | 3263.14 | **0.07** |

AIC=Akaike Information Criterion; BIC=Bayesian Information Criterion; cAIC=consistent Akaike Information Criterion; ssBIC=sample size adjusted Bayesian Information Criterion; CLC=Classification Likelihood Criterion; ICL BIC=Integrated Completed Likelihood Criterion with BIC approximation; NEC=Normalized Entropy Criterion

*Bolded values indicate the class soalution most supported by each criterion.

**eTable 2. Class solution criteria for male Medicaid beneficiaries who died of suicide (n=175). A two-class solution was selected.**

| **Class solution** | **Parameters** | **Log likelihood** | **Entropy*** | **AIC*** | **BIC*** | **CAIC*** | **ssBIC*** | **CLC*** | **ICL BIC*** | **NEC*** |
| --- | --- | --- | --- | --- | --- | --- | --- | --- | --- | --- |
| **1** | 19 | -1826.59 | 0.00 | 3691.19 | 3751.32 | 3770.32 | 3691.15 | 3653.19 | 3751.32 | 1.00 |
| **2** | 39 | -1723.80 | 24.84 | 3525.60 | **3649.02** | **3688.02** | 3525.52 | 3497.28 | **3698.70** | 0.24 |
| **3** | 59 | -1684.22 | 21.52 | 3486.44 | 3673.16 | 3732.16 | 3486.33 | 3411.48 | 3716.20 | 0.15 |
| **4** | 79 | -1653.17 | 20.72 | 3464.35 | 3714.37 | 3793.37 | 3464.20 | 3347.79 | 3755.81 | 0.12 |
| **5** | 99 | -1623.15 | **30.23** | 3444.30 | 3757.61 | 3856.61 | 3444.11 | 3306.76 | 3818.07 | 0.15 |
| **6** | 119 | -1596.22 | 27.63 | **3430.44** | 3807.05 | 3926.05 | **3430.22** | 3247.70 | 3862.31 | 0.12 |
| **7** | 139 | -1577.85 | 22.44 | 3433.71 | 3873.61 | 4012.61 | 3433.44 | **3200.59** | 3918.49 | **0.09** |

AIC=Akaike Information Criterion; BIC=Bayesian Information Criterion; cAIC=consistent Akaike Information Criterion; ssBIC=sample size adjusted Bayesian Information Criterion; CLC=Classification Likelihood Criterion; ICL BIC=Integrated Completed Likelihood Criterion with BIC approximation; NEC=Normalized Entropy Criterion

*Bolded values indicate the class solution most supported by each criterion.
